# The use of educational technology for interactive teaching in lectures

**DOI:** 10.1016/j.amsu.2021.01.051

**Published:** 2021-01-21

**Authors:** Faiz Tuma

**Affiliations:** Department of General Surgery, Central Michigan University College of Medicine, 912 S. Washington Avenue, Suite #1 Saginaw, MI, 48601-2578, USA

**Keywords:** Educational technology, Interactive learning, Medical education, Lectures

## Abstract

Students often feel overwhelmed by the volume and complexity of knowledge and skills required to learn. Along with this challange, educational technology has been gradually introduced in medical education to facilitate learning and improve outcomes. It became an essential part of communication, storing and transferring information, audio-visual media use and production, and knowledge sharing. Technology's role has been expanding from a mere tool of study and inquiry to an approach and integrated use in education. Its use in medical education is continuously evolving. However, the impact and optimal use of various technology applications are not clearly defined. There are multiple challenges facing educators to choose the right application for the specific educational purpose. Hence, studies and evaluation reviews are needed to inform the better-defined use of educational technology.

This review aims to discuss and evaluate various educational technology applications in medical education, focusing on interactive learning during lectures. Lectures and other group learning sessions are common activities used by medical schools. Promoting interactive learning in large groups is known to be challenging. The advances in technology to facilitate communication and promote interaction is a promising adjunct for lectures interactivity.

## Highlights

• Interactive teaching is a fundamental approach to enhance learning in medical education.
• Lecturing is still one of the most commonly used teaching methods, mainly due to its practicality and convenience for conveying information to large groups of learners using limited educational resources.
• Learning theories are used to build the skeleton of the education processes and activities.
• Technology's role has been expanding from a mere tool of study and inquiry to an approach and integrated use in education.
• Technology can enhance interactive learning in well-designed and structured formats based on educational objectives.

## 1 Introduction

Medical education is a complex and challenging process involving acquiring a large volume of knowledge and skills and can be highly stressful [1]. Medical students often feel overwhelmed by the high amount of knowledge required to learn [2]. They often have difficulties in retaining factual information [3]. Maintaining up-to-date knowledge and adopting evidence-based knowledge and lifelong learning are essential [4]. Improving the efficiency of educational activities, including lectures, is essential. Many researchers have explored methods of maximizing learning efficiency [2]. Searching for better medical education strategies continues, and it shares the common theme of increasing interest and attention [5]. Multiple approaches have been used to promote students’ interaction. Interactive learning represents one of the fundamental approaches to enhance learning in medical education. This article aims to review the role of lectures in medical education and the introduction of educational technology to enhance interactive learning in lectures.

### 1.1 Lectures in medical education

For many decades, lectures have been considered one of the most common teaching methods in medical education [6]. The lecture's simple structure, which involves delivering information by a teacher talking directly to a group of gathered learners, makes it easy to use. Lecturing is still one of the most commonly used teaching methods, mainly due to its practicality and convenience for conveying information to large groups of learners using limited educational resources [6]. Some lectures result from significant preparation, perhaps using additional modalities such as video, yet many remain a one-way delivery of a topic content to the audience [7]. The literature reveals many advantages to explain their persistence. They can be an effective way to transmit factual knowledge to a large number of students [8]. They are a practical and efficient use of the resource (one educator for a large group of students, one room to organize) [9]. They can be used to vitalize ideas, provide up to date, and a large amount of information, supplement textbooks, provide opportunities to clarify information, and save students the search to find information [10].

### 1.2 Interactive learning in lectures

Enhancing involvement, participation, and maximum interactivity from both the students’ and the faculty is an area of potential improvement in medical education [11]. There is an increasing trend toward shifting from traditional teaching to student-centred teaching that actively engages students [12]. Improving the educational activities setting from the traditional - almost one-sided (speaker) activity - to both sides (speaker and learners) participating in an interactive, entertaining, and higher learning outcome activity can enhance the educational activity for better outcomes [13].

There are potentially multiple difficulties in fostering interactive learning during lectures. Educators and educational institutions have been facing growing challenges to accommodate a large number of students in the lectures and maintain a high level of interactions and student satisfaction [14]. Achieving interaction in lectures with a large number of students is difficult [15]. The need for a practical and efficient way to achieve interaction is of high importance. Enhancing engagement and interaction during lectures is one of the fields of work to transform learning through lectures. Teachers have practiced the use of questions as a tool to enhance interactivity to facilitate the learning process [16].

Educators need to use the learning theories principles in both the classroom process and content to improve teaching effectiveness [17]. The pertinent theories' description is reviewed briefly here to support understanding the move from inactive to active lectures. Learning theories are used to build the skeleton of the education processes and activities.

### 1.3 Theoretical underpinning

Learning theories fall within five main categories: 1- cognitive learning theory, 2- behavioural learning theory, 3- constructivism learning theory, 4- connectivism learning theory, and 5- adult learning theory. The two most theories will be discussed here to understand better using educational technology to achieve educational goals.

1 **The Cognitive learning theory.** Cognitive theory is used to provide demonstrations, stimulate mental processing of information, and detail real-world scenarios, and instructors are advised to create appropriate instructional activities to help learners effectively and efficiently create their knowledge [18]. Cognitive learning theory explains the mental process of deeper thinking and learning in response to the various questions and interactions implemented by technology. When responding to interaction, learners are likely to be motivated to understand their learned materials in depth.
2 **The Constructivism learning theory.** This theory is outlined by Lev Vygotsky (1896–1934) [19]. Constructivism theory defines the learning process in social interaction, language, and cultural aspects [20]. Learning in medical education is enhanced by interaction. The most appealing learning technique for human beings is interaction [21]. Therefore, this theory makes a common basis for conducting education research [22]. The constructivist learning approach offers students advice, guidance, and inspiration [23]. Students in group learning activities can actively construct their knowledge using the new information and incorporating it in their existing schema of knowledge. Learning strategy can be effective using the constructivist approach that utilizes common classroom interactions; students are inspired and challenged using the constructivist approach [24]. Interactivity and engagement are essential for adult and constructive learning. Interactivity is an essential concept in Constructivism, and the quality of learning is higher with engagement [21].

Students' passivity in lectures has been identified as a weakness [25]. However, lectures were still an effective teaching method when given as large-group interactive learning sessions with discussion and frequent questions to students who have prepared in advance [26]. For more effective learning, educators need to change their lecture time to incorporate methods and techniques to facilitate students' participation, communication, and collaboration for learning, with simultaneous evaluation of these methods' effectiveness on their learning [6]. Interactive didactic lectures teaching theoretical knowledge involving students actively within the lecture time and activity was considered a more effective learning tool [27]. During the lecture, learners' interaction, and participation are activities of active adult learning where adults construct their learning actively. In an interactive lecture, learners are asked to engage actively and process knowledge throughout the lecture's activity, and they take an active part in structuring the content and directing the focus of the lecture towards areas that fulfill their needs [28].

It has been proposed that using teaching methods that promote active participation and encourage self-directed learning can enhance delivering core knowledge and explaining difficult concepts leading to better learning outcomes [29]. The active learning approach, which requires students to actively engage with learning materials, participate in the class, and collaborate with other classmates, is considered the most effective approach for efficient teaching [30]. Therefore, educators should use the lecture to encourage students to build their knowledge, relationships and enhance the application of knowledge by selecting the right learning approach [24]. Teachers and students express a positive attitude toward interactive and team-based learning [31]. Facilitating the group interaction in learning activities needed both educators' extra skills and tools of sharing and interactions. Technology, with various tools and applications, provided significant support and facilitation to education and learning. Educational technology is an essential adjunct to education in the current time.

### 1.4 Educational Technology

Education technology is undergoing significant changes due to computers' increasing pervasiveness [32]. Technology is an essential part of communication, storing and transferring information, audio-visual media use and production, and sharing. Technology's role has been expanding from a mere tool of study and inquiry to an approach and integrated use in education [33]. Educators have a responsibility to guide their students to the proper and advantageous use of educational technology. Technology has invaded and made life better in many ways; however, it can constitute a severe threat to the physical and mental well-being [34]. Therefore, with the digital age, technology is continuously changing, requiring careful and cautious monitoring to direct use.

### 1.5 Educational technology in medical education and lectures

The use of educational technology in medical education is continuously evolving. Educators are integrating new technology into the medical curriculum; however, newer technology's exact impact on educational outcomes remains unclear [35]. There are challenges in the practical use of interactive learning technology despite sufficient research in the field [36]. Unfortunately, the evidence suggests that technology is often poorly integrated with other educational activities [37]. There is an urgency to develop and apply interactive technology in higher education [36].

Recent developments in educational technology can revolutionize medical education [38]. There is a compelling need to incorporate the most current technology in education, and Health Information Technology (HIT) is considered critical to medical education, raising the call to develop HIT competency [39]. Technology can produce substantial educational benefits [40]. Educators may use these new technologies effectively to transform learning activities into personalized, interactive, and collaborative experiences [41].

Educational systems have integrated numerous technologies such as computers, smartphones, tablets, and cloud-based services, each involving modifying the instructional strategies and teaching methods [42]. There are several reasons to incorporate technology within the classrooms for teachers, such as easier storage of materials, efficient communication, and portability of the teaching objects. Many have embraced new technologies to enhance the overall learning process [43]. Their use varies according to the need and specific situations and purposes. Some of the key factors in increasing technology's popularity are mobility, ease of access to Information, cost efficiency, better communication means, wide applications, and timesaving [44]. Other fields of professions may have additional perspectives and motives for the prevalent use of technology. The technology acceptance model (TAM) framework helps understand users' adoption of technologies, especially in the workplace [45]. TAM highlights that people's use of technology depends on their perception of usefulness and ease of use. Applying learning and educational technology also needs to invoke learning users' intended impact [46].

Medical, educational technology can be effectively used in a various medical education setting [47]. Educational technology must provide various means of delivering education [48]. Multiple technologies are already being used in medical education [41]. Educational technology has revolutionized the delivery and impact of education [49]. Examples of some of the educational technology tools that are widely used are: web-conferencing or webinars, video lecture capture technology (VLCT) for asynchronous activities, Course Management Systems (CMS) or platforms to organize the course interactions, wikis as a collaborative work to simulate group work, podcasting (a digital audio file shared via the web), real simple syndication (RSS) (system for distributing content from an online publisher to subscribed users), simulation in training, and digital learning objects (collection of digital content, including practice and assessment items combined into a single learning object) [[48], [49], [50]]. Polling and various quiz platforms became easily and efficiently used in lecture and other educational activities.

Lectures are used in most medical schools to teach large groups efficiently. They convey up-to-date information, clarify concepts, and guide students learning. Hence, fostering the quality of lectures will impact educational outcomes positively. Interactive learning in lectures can be enhanced with educational technology applications in a well-designed and structured format. Educators should choose the appropriate technology tool based on their educational needs and objectives. Creating interactivity within the classroom is becoming easier with educational technology tools [51]. Tools that facilitate learner's participation and engagement, such as the audience response system (ARS), are effective in several aspects. The ARS is a widely used simple technology to enhance interactive learning and makes classroom teaching more student-centred while creating an interactive environment driven by questions-based instructions [52].

The ARS is a system of instant feedback that can be integrated into the lecture or presentation. It can be used via a student's mobile device or small, dedicated hand-held, remote keypads to respond to questions posed by the educator. Questions of different types and various educational purposes are integrated and used to allow for audience feedback and interactions. ARSs have been used in different ways, including a learning strategy to facilitate increased attention, interaction, instruction, student preparation, and discussion; to record attendance and participation; to provide formative and summative knowledge assessments; and provide diagnostic information tutor [53]. The technology has been successfully used in various course formats, ranging from optional tutorials [54] to formal standard lectures and cooperative learning through peer instruction [55]. Students and educators who have used ARS were enthusiastic and found it improving student learning [56].

The increased use and interaction with multimedia content is vital to improve learners' learning outcomes, which led to increased use of video lectures to increase interactivity [57]. Interactive videos and other media have been used in various formats to enhance information delivery and stimulate interactivity at several levels. It also enriches the diversity of activities within the educational curriculum. Through interactive video lectures, engagement can be increased with rich learner-content [58]. Animation, as another type of media is a powerful medical education tool that can be designed solely for the learning objectives. Integrating interactive components in animation is part of creating the animation product and has been used to promote interactive learning—the content, whether texts or media, provide the materials for discussions and interactions. While learners’ input or interaction make are the tools for interactive learning. Therefore, integrating rich materials and well-selected interactive tools in alignment with the educational objectives can improve leering outcomes.

The strategic use of entertainment for educational purposes has been commonly used [59]. Using entertainment in education has several advantages but is also associated with downsides like rewarding for mastery, not efforts, poor learning from failure, and the weak role of feedback [60]. Depending on the educational setting, type of entertainment, and the specific purpose, entertainment can be designed. The efficiency of interactive educational technology depends on the participants and their prospects [36]. Interactive educational technology may positively affect students’ mastery of learning and significant developing and nurturing effect [36]. Well-established infrastructure for technology integration promotes easier and efficient use [61].

### 1.6 Future perspectives

The rapid advancement and increasing applications of technology make predicting future directions challenging. However, successful application experiences and rising educational needs based on learning theories provide practical and insightful guidance. Focusing on the process of learning and teaching more than providing resources will be the next priority in the mission of education. Abundant and diverse learning resources (digital books, articles, videos, podcasts, and other learning materials) have become widely available, transferable and shareable with the advanced technology. While teaching and learning processes such as supervising, feedback providing, and individualized learning are still at the early stage of technology use. Many of the currently uses of technology are attempts to simulate the tradition teaching with newer tools. This use is beneficial but of limited value compared to highly interactive, learners and objectives customed, and autonomy or independency driven educational activities. Activities like asynchronous learning and interacting, automated feedback enhanced learning, and imagery provoking educational activities are expected to prevail and show effective educational outcomes.

The use of technologically enhanced educational activities has not been fully studied [62]. Thus, studies are essential to evaluate the appropriate educational tool to achieve high demands on better and interactive educational activities. More research is needed to develop education programs, specific educational tools, and guidelines for use and integration in the curriculum [63]. Further research into the use of technology in different practice types would differentiate technology-related training, which could help develop the best practice models for medical education technology [64].

## 2 Conclusions

Educational Technology has been integrated into multiple aspects of medical education. The use and integration of technology should be directed by the educational needs to optimize the learning outcomes. Specific role and objectives pre-requisites for optimal results. Fostering interactive learning in educational activities can be achieved with selected technologies. Various technology applications have been used to enhance learners’ engagement and higher participation in lectures and other group learning sessions. Collaborative and continuous efforts are required to identify or create the appropriate technology tools for efficient education based on educational theories.
